# Trional

**Published:** 1895-04

**Authors:** 


### Selections and Abstracts.

Trional.—Claus (*International klinische Rundschau*, No. 45, 1894) speaks highly of trional in the insomnia of children. He believes that it should be avoided in the insomnia of organic brain diseases, as meningitis, etc., but finds it especially useful in chorea, convulsions, and night-terrors. Trional is of little service in insomnia due to pain. The doses used by the author were 0.2 gramme to 0.4 gramme for infants from 1 month to 1 year, increased to 1.2 to 1.5 grammes for children from 6 to 10 years. In the insomnia due to toxic influence chloral is more effective.

